# Adherence to treatment regimen and bleeding rates in a prospective cohort of youth and young adults on low-dose daily prophylaxis for severe hemophilia A

**DOI:** 10.1186/s12878-016-0067-3

**Published:** 2016-11-08

**Authors:** Terry Mizrahi, Jean St-Louis, Nancy L. Young, Francine Ménard, Nichan Zourikian, Evemie Dubé, Georges E. Rivard

**Affiliations:** 1 CHU Sainte-Justine, University of Montreal, 3175, chemin de la Côte-Ste-Catherine, H3T 1C5 Montreal, Quebec Canada; 2 Laurentian University, Sudbury, Canada

**Keywords:** Hemophilia A, Prophylaxis, Health-related quality of life, Annualized bleeding rates, Daily dose, Adherence

## Abstract

**Background:**

When availability and/or affordability of anti-hemophilic factor concentrates are limited, optimal prophylaxis regimens in severe hemophilia A (HA) remain to be determined. In selected situations, low-dose daily prophylaxis (LDDP) may be an effective and economical option. The goal of our study was to evaluate if subjects on a LDDP regimen could achieve adherence and good clinical outcome.

**Methods:**

Seventeen subjects (age between 15.2 and 28.4) on LDDP suffering from severe/moderate HA were followed prospectively for 2 to 3 years as part of a health-related quality of life (HRQoL) study. Bleeding and treatments data were collected using electronic diaries and validated every three months. The SF-36 questionnaire was administered at the beginning of the study and then every 6 months until the end of the study.

**Results:**

The subjects (mean age 22.0, median 21.9, standard deviation 4.06), were all from a single centre and on LDDP for at least 12 months as part of their routine care before entering the study. Fifteen subjects were prescribed a daily dose of 500 IU factor VIII (FVIII) and 2 subjects received 1000 IU FVIII per day, resulting into a median dose of 7.1 IU/kg/day (ranging from 4 to 13 IU/kg/day) and of 2591 IU/kg/year. Median adherence (the percentage of the prescribed daily dose received) was 84 % (mean 80 %, range 57 % to 94 %) throughout the study. Seventy-six bleeds in the 6 index joints and 51 other types of bleeds were observed throughout the study. The median annualized bleeding rate in joints (ABRjoints) was 0.7 and the median annualized bleeding rate for all bleeds (ABRall) was 1.6. The Physical Component and Mental Component Summary scores of SF-36, and the Hemophilia Joint Health Score were not significantly different over the course of the study (respective medians of 49.8, 52.4 and 16.0 at entry; vs. 52.5, 51.5 and 16.0 upon exit).

**Conclusions:**

This prospective longitudinal study in youth and young adults shows that LDDP may be associated with low ABRs, adequate adherence and HRQoL comparable to previously reported.

## Background

Hemophilia A (HA) is an inherited X-linked bleeding disorder leading to recurrent joint bleeding and chronic arthropathy, if untreated. Prophylaxis (i.e. regular, long-term anti-hemophilic factor replacement) is recommended as a standard of care by organizations such as the World Federation of Hemophilia, for treating severe hemophilia [1]. Based on reports of the 25-year Swedish experience [2], 3-times-weekly prophylaxis was established in the 90’s as the standard, to help maintain adequate plasma factor VIII (FVIII) trough levels (>0.01 IU/mL), and prevent spontaneous joint bleeding in severe HA. However, breakthrough bleeding still occurs in certain subjects with severe HA, despite standard thrice-weekly prophylactic doses, perhaps due to variations in FVIII trough levels. Based on pharmacokinetic principles, more frequent infusions of FVIII at lower doses may result in comparable or improved trough levels [3, 4]. A 2012 pilot study demonstrated that daily prophylaxis is feasible, with a substantial reduction (30 %) in factor concentrate utilization - albeit with a higher bleed rate in certain patients, and a decreased quality of life [5]. Verma et al. recently demonstrated that a very low dose, twice-weekly prophylaxis regimen in children was effective at lowering bleed rates compared to on-demand treatment [6].

The present study describes clinical outcomes, health-related quality of life (HRQoL) and adherence to treatment in a cohort of youth and young adults with severe HA from a single centre, followed for 2 to 3 years on a Low-Dose Daily Prophylaxis (LDDP) regimen. This cohort was part of a larger, prospective and longitudinal study of HRQoL in Canada, the Canadian Helixate Quality of Life Study (CHeQoLS).

## Methods

CHeQoLS was a prospective non-interventional study describing longitudinal HRQoL in 48 youth and young adults with moderate or severe HA [7], conducted over three years in 6 Canadian Hemophilia Treatment Centres (HTCs). Male subjects 14 to 29 years of age were eligible for CHeQoLS if they had moderate (FVIII level 0.02 – 0.05 U/ml) or severe HA (FVIII level <0.02 U/ml) and were using the antihemophilic factor concentrate Helixate FS^®^ (CSL Behring, King of Prussia, PA, USA) as a prophylactic or on-demand treatment. Exclusion criteria were presence of a current inhibitor to FVIII (defined as ≥ 0.6 Bethesda Units/mL), human immunodeficiency virus (HIV) or symptomatic hepatitis C virus (HCV) infection [7]. CHeQoLS was funded by CSL Behring Canada and was registered in Clinical Trials.gov under the registry number NCT01034904 on December 17, 2009.

One of the 6 CHeQoLS study sites, CHU Sainte-Justine in Montreal, had introduced LDDP as an alternative treatment regimen in 1998, in an effort to reduce frequency of hemarthroses in patients who manifested breakthrough bleeds, while on standard 3-times-weekly FVIII prophylaxis. This regimen was well tolerated and became the regular or routine treatment regimen for many patients at this HTC, irrespective of the presence of breakthrough bleeding. None of the other CHeQoLS participating sites had patients on LDDP.

The current study is a secondary analysis of all 17 subjects participating in the CHeQoLS who were specifically from CHU Saint-Justine, and who were on a LDDP regimen as part of their routine care when they were recruited in CHeQoLS. This secondary analysis is in line with the original intent of the CHeQoLS study and its scope was included in the written informed consent provided by all subjects prior to enrollment. The consent procedure is described below in the Declarations section. Permission to conduct this analysis was granted by the owners of the data.

A medical history was obtained at baseline (including hemophilia severity, current treatment, concomitant medical conditions, target joint history in the year prior to enrollment and history of previous surgeries and major bleeding events) as well as a comprehensive demographic survey in relation to educational and professional experience. Bleeding and treatment logs were recorded by subjects using the HeliTrax^®^ (CSL Behring, King of Prussia, PA, USA) electronic diary, and later reviewed and validated on a quarterly basis by the study nurse. A physical examination, including joint assessment using the Hemophilia Joint Health Score (HJHS) 2.0 [8] was performed by the study physiotherapist at baseline and yearly while on study. The HJHS assesses 6 key joints (elbows, knees and ankles) for swelling, muscle atrophy, strength, crepitus, range of motion, pain and gait, resulting in a total score ranging from 0 to 124 – where the absence of abnormal findings corresponds to a score of 0.

Annualized bleeding rate for all bleeds (ABRall) and for joint bleeds (ABRjoints) were calculated based on electronic diary records obtained with HeliTrax^®^ by computing the absolute number of bleeds for each successive 12 month period of observation. Adherence to treatment was expressed as a percentage, calculated by dividing the number of prophylactic infusions administered (based on electronic diary records) by the number of infusions prescribed over a given period of time.

A generic HRQoL questionnaire widely used in hemophilia, the Medical Outcomes Study 36-Item Short Form (SF-36v2), was completed at baseline and every six months by all study subjects. SF-36v2 consists of 36 questions from which scores are computed in 8 domains that can be summarized in 2 summary scores: the Physical Component Summary (PCS) and the Mental Component Summary (MCS) scores. These summary scores are norm-referenced, with an expected mean of 50 and a standard deviation of 10 points in a normal population [9].

The data collected were analysed primarily using descriptive statistics. Parametric statistics were used for normally distributed variables, and non-parametric statistics were presented for data whose distribution was non-normal. A paired-sample t-test (two-tailed) was used to compare HRQoL scores at study entry and exit.

## Results

During CHeQoLS recruitment, 39 potential subjects aged 14 to 29 years with moderate or severe HA were registered at the CHU Sainte-Justine HTC. Fourteen subjects were excluded because they did not provide consent or meet all inclusion criteria, received treatments other than Helixate FS^®^, or were unable to comply with data collection (electronic diary) or to fill questionnaires in French/English. Of the 25 CHU Sainte-Justine subjects recruited into CHeQoLS, 17 subjects (mean age 22.0, median 21.9, standard deviation 4.06) were on LDDP and are included in this study. All subjects had severe/moderate HA, had been on LDDP at least a year prior to recruitment and remained on a LDDP regimen throughout the study. Other characteristics of study subjects are summarized in Table 1.

**Table 1:** Study cohort characteristics at baseline

Sample Size	17
Ages (years)	
mean	22.0
median	21.9
range	15.2–28.4
Body Mass Index (kg/m^2^)	
mean	25.2
median	24.5
range	18.4–37.7
Race	All Caucasians
Type of hemophilia	16 severe
	1 moderate
≥1 Target Joint	24 %
Daily factor concentrate dose	500 IU daily = 15
	1000 IU daily = 2
Education	17.6 % college level
	17.6 % secondary level
	11.8 % university level
	53.0 % other/unknown
Occupation	47 % students
	35.3 % high physical demanding occupation
	11.8 % low physical demanding occupation
	5.9 % other/unknown

The prescribed dose for LDDP was 500 IU per day in 15 cases and 1000 IU per day in 2 cases. This translated into a median daily dose of 7.1 IU/kg/day (range 4–13 IU/kg/day), equivalent to 2591 IU/kg/year (range 1460–4745 IU/kg/year). Adherence of individual subjects to their LDDP regimens ranged from 56 % to 98 % (median 85 %, mean 81 %) over their entire observation period. Adherence rates remained relatively stable for the cohort throughout the course of observation, ranging from 58 % to 99 % (median 88 %, mean 82 %) during the first three months of observation, to 48 % to 100 % (median 80 %, mean 77 %) during the last three months of the study.

Over the 516 subject-months of cumulative observation, there were 76 hemarthroses in the 6 index joints and 51 other types of bleeds. The median ABRjoints and ABRall values were respectively 0.7 (range 0.0–9.6) and 1.6 (range 0.0–11.8) (Table 2). The median HJHS was 16 (range 0–34) at study entry, and remained unchanged upon exit (median 16, range 0-38) (Table 3). The mean PCS and MCS scores of the SF-36v2 respectively were 49.1 (SD = 5.65) and 51.0 (SD = 7.83) at study entry, vs. 51.8 (SD = 5.92) and 47.9 (SD = 9.45) upon study exit (Table 3). The minimally important difference (MID) threshold for the SF-36v2 was used as the criterion for clinical significance [9]. The minimally clinically relevant differences are respectively defined as 3 and 2 for PCS and MCS scores [9]. The differences between the scores at entry and exit approached the MID, but were not statistically significant (PCS *t* = –1.68, *P* = 0.113; MSC *t* = 1.75; *P* = 0.099).

**Table 2:** Summary of Annual Bleeding Rates (ABRs) for index joints (ABRjoints) and all bleeds (ABRall) in 17 male subjects with severe/moderate HA on a LDDP regimen

	Annualized bleeding rates in joints (ABRjoints)				Annualized bleeding rates for all bleeds (ABRall)			
	Year 1	Year 2	Year 3	Average of 3 years	Year 1	Year 2	Year 3	Average of 3 years
Median	1.10	1.00	0.00	0.70	2.00	2.00	0.90	1.63
Minimum	0.00	0.00	0.00	0.00	0.00	0.00	0.00	0.00
Maximum	11.50	9.20	8.00	9.57	11.50	13.80	10.00	11.77
Standard Deviation	2.68	2.47	2.81	2.65	3.57	3.34	3.67	3.53

**Table 3:** Description of SF-36v2 and HJHS scores upon study entry and exit in 17 male subjects with severe/moderate HA on a LDDP regimen

	PCS		MCS		HJHS	
	Baseline	End of study	Baseline	End of study	Baseline	End of study
Median	49.8	52.5	52.4	51.5	16.0	16.0
Minimum	40.0	40.7	33.6	32.6	0.0	0.0
Maximum	59.2	61.0	63.0	62.2	34.0	38.0
Mean	49.1	51.8	51.0	47.9	14.4	13.9
Standard Deviation	5.66	5.92	7.83	9.45	8.54	8.98
*t*-test	-1.68		1.75		0.45	
*P* value	0.11		0.10		0.66	

## Discussion

Primary prophylaxis is considered to be the gold standard for preserving joint function in severe HA. This recommendation is supported by many studies, notably the Manco-Johnson et al. randomized and controlled trial, comparing on-demand therapy to full-dose prophylaxis [10, 11]. Secondary prophylaxis in children, adolescents and adults with HA has also been shown to be beneficial for joint protection [12–17]. In addition, prophylaxis prevents bleeding and reduces the risk of life-threatening hemorrhages [10, 11, 18]. The purpose of our study was to describe clinical outcomes (bleeding rates and joint health), adherence to treatment and HRQoL in a cohort of patients treated on a LDDP regimen, while participating in a non-interventional and longitudinal study of youth and young adults with moderate or severe HA in Canada [7].

In our study, LDDP resulted in a median ABRall of 1.6 and a median ABRjoints of 0.7 with a tendency towards a reduction in bleeding between the first year and the third year (Table 2), but with no difference in joint health status as evaluated by the HJHS (Table 3). Our ABRs are similar to what has previously been described in the literature [11, 14]. Valentino et al. compared the efficacy of two prophylactic regimens (standard 20–40 IU/kg every other day, or pharmacokinetic-tailored 20-80 IU/kg every third day) to on-demand treatment in 7- to 59-year-old male subjects. ABRall in both prophylactic regimens were comparable and statistically less than on-demand therapy (respectively 1.0, 2.0 and 43.9 for standard, pharmacokinetic-tailored prophylaxis and on-demand therapy). In addition, median ABRjoints was higher for on-demand treatment than prophylactic treatments (38.3 vs 1.0) reflecting the benefits of long-term prophylaxis [11].

Discontinuation or inadequacy of prophylaxis can result in increased bleeding episodes and/or worsening of target joint status [18–20]. Non-adherence and interference with lifestyle are considered the main causes for discontinuing prophylaxis [18], while prophylaxis efficacy may be compromised by previous joint damage or unfavourable pharmacokinetics [21]. Our results showed adequate overall adherence throughout the study duration (median 85 %, mean 81 %), with little difference between the first three months vs. last three months of the study (respectively median 88 %, mean 82 %, vs. median 80 %, mean 77 %). Previous studies have reported average prophylaxis adherence in severe hemophilia A or B of between 58.8 % and 87 % [22, 23]. In a study of 31 adults patients with severe hemophilia A or B, Ho et al. [24] reported adherence to a prescribed prophylaxis frequency (3 times per week) and dosage (2000 UI/dose) to be respectively, 76 % and 93 % on a 78-week period, which was similar to our results. Moreover adherence is positively correlated with younger age and longer exposure to prophylaxis [23, 24], a finding also illustrated in our study, as patients were on a LDDP regimen for at least a year before enrollment.

Long-term prophylaxis in severe HA can however be limited by cost issues. Fischer et al. reported a 66 % higher total cost in high-dose vs. intermediate-dose prophylaxis in children and adults, due to a higher FVIII consumption (4000 IU/kg/year vs 2100 IU/kg/year) [25]. Unsurprisingly, Valentino et al. observed a lower median annualized consumption for on-demand treatment compared to prophylaxis, respectively 2152.2 and 5733.3 IU/kg per year [11]. Interestingly, in our cohort, the LDDP regimen also resulted in a lower median FVIII consumption compared to 2 previous reports for standard prophylactic regimens (2591 IU/kg/year in our study compared to 3223-4082 IU/kg/year in respectively ≥19 and 13-18 year old subjects [26]; 3664.5–3795.8 IU/kg/year in respectively 12-25 and 26–55 year old subjects [14]). Crivaniu-Gaita et al. also found once-a-day prophylaxis was associated with a 23.5 % decreased FVIII consumption in youth and young adults with severe HA, compared to standard prophylactic regimens [27]. A relatively lower annual consumption of FVIII concentrate associated with LDDP may reduce the annual cost per patient if not associated with increased bleeding.

Given the chronicity, potential chronic morbidity and the burden of treatment in severe HA, HRQoL becomes a valuable assessment outcome in this population. Although certain disease-specific tools have been established to measure HRQoL in hemophilia [28], the generic instrument SF-36 has been widely used in HA. SF-36 also allows comparison with normal population or with groups with other diagnoses. In our cohort, the PCS and MCS summary scores of SF-36v2 varied minimally from baseline to the end of study and were similar to those reported at baseline in the larger CHeQoLS study (mean PCS 51.7 ± 6.5, mean MCS 51.1 ± 8.6) [7]. This low variability could be explained by the fact that our subjects were already on a LDDP regimen at study entry, but also by the low number of subjects enrolled in our study. In the Advate PASS study, Klamroth et al. assessed HRQoL in HA patients compared to healthy subjects, and to populations with other chronic diseases [29]. In their study, the PCS was reduced in HA (42.3 ± 0.77) compared to age-matched healthy subjects (54.5 ± 0.24) or those with chronic back pain (46.9 ± 0.4), but comparable to subjects with rheumatoid arthritis (43.6 ± 1.89). Age was an important factor across all populations, as the PCS generally decreased over the years. In our study, mean MCS was unchanged at the end of the study (51.5 ± 9.45 compared to 52.4 ± 7.83) but still higher than the US general norms (47.7 ± 0.24) reported by Klamroth et al. [29], whereas mean PCS was comparable to baseline (52.5 vs 49.8) and US general norms (50.7 ± 0.23).

The major limitations of our study are a result of our small sample of subjects, all of whom were from a single HTC, and the lack of a control group. The treatment philosophy and type of ancillary patient support of a particular HTC may not be possible in all settings. While there were subjects from CHU Sainte-Justine that were not on LDDP, this sample was small (8) and not comparable to our LDDP group.

## Conclusions

In this small cohort of youth and young adults, we observed that LDDP can be associated with good overall adherence to treatment, low ABRjoints and ABRall, stable joint status, and SF-36 scores comparable to those reported in the literature for severe HA. Furthermore the relatively low annual consumption of FVIII concentrate means that LDDP may reduce the annual factor concentrate cost per patient. This may be an important economic consideration in lower-income countries.

## Abbreviations

ABR: Annualized bleeding rate
CHeQoLS: Canadian helixate quality of life study
FVIII: Factor VIII
HA: Hemophilia A
HJHS: Hemophilia joint health score 2.0
HRQoL: Health-related quality of life
HTC: Hemophilia treatment centres
IU: International units
LDDP: Low-dose daily prophylaxis
MCS: Mental component summary
MID: Minimally important difference
PCS: Physical component summary
